# Age Diversity Climate Affecting Individual-Level Work-Related Outcomes

**DOI:** 10.3390/ijerph19053041

**Published:** 2022-03-04

**Authors:** Lara Bellotti, Sara Zaniboni, Cristian Balducci, Luca Menghini, David M. Cadiz, Stefano Toderi

**Affiliations:** 1 Department of Psychology, University of Bologna, 47023 Cesena, Italy; sara.zaniboni4@unibo.it (S.Z.); cristian.balducci3@unibo.it (C.B.); luca.menghini3@unibo.it (L.M.); stefano.toderi@unibo.it (S.T.); 2 Department of Management, Technology and Economics, ETH Zürich, 8092 Zürich, Switzerland; 3 The School of Business, Portland State University, Portland, OR 97201, USA; dcadiz@pdx.edu

**Keywords:** age diversity climate, intentions to quit, job-related wellbeing, work engagement

## Abstract

The present study answers the call for more studies to investigate the age diversity climate’s effect on individual-level outcomes. Building on the social identity approach and social exchange theory, we surveyed 110 Italian employees aged between 18 and 61 years old (*M* = 46.10, *SD* = 10.02) and investigated the role of age diversity climate in predicting intentions to quit (H1), job-related wellbeing (H2), and work engagement (H3). Our findings confirmed the hypotheses (H1 and H2), showing the added effect of age diversity climate over and above age, job tenure, role clarity, job demands, job control, perceived support, and perceived job and organizational fit. In fact, age diversity climate accounted for a significant increase in the variance explained for two of the three hypothesized models (i.e., intentions to quit and job-related wellbeing, but not work engagement). To conclude, this study contributes to the existing literature by showing the age diversity climate’s predictive value for turnover intentions and job-related wellbeing, and corroborating the importance of supporting age diversity through a variety of Human Resources Management strategies.

## 1. Introduction

Over the last few decades, the workforce has become increasingly age diverse, resulting in a broader range of younger and older individuals working together like never before. A significant factor of the increasing age diversity is that more people are working later into their lives. In fact, most Western industrialized societies have been increasing the retirement age to qualify for pensions, extending people’s working life [1]. As a consequence, employment rates of people aged 55+ have been increasing in most OECD countries, jumping from 44% in 2000 to 60.4% in 2020 [2]. 

Within the workplace, diversity refers to those visible (e.g., age, gender) or invisible (e.g., sexual orientation) characteristics of employees that can either be a resource or an obstacle to the achievement of today’s organizational goals of competitiveness and performance [3,4]. Accumulating research in the field of age at work indicate that a potential obstacle stemming from increased age diversity within the workplace is the rise of age prejudice and age discrimination [1,5,6,7,8]. In fact, research supports that the emergence of positive or negative effects of diversity largely depends on the workplace practices that encourage or devalue certain personal characteristics (e.g., age) [4,9]. 

Age prejudice and age discrimination are pervasive phenomena characterized by negative attitudes and behaviors towards individuals of different age [7,10]. As suggested by the social identity approach [11,12,13], when age diversity is high, the salience of age increases and this is likely to increase employees’ self-categorization processes based on age, resulting in identification with members of their own age group and discrimination towards members of a different age group [1,11,13,14]. In this scenario, age diversity will translate into negative organizational (e.g., increased workplace conflict) and individual outcomes (e.g., reduced wellbeing) [5,6,7]. Indeed, poor diversity management and/or improper workplace practices can elicit additional challenges associated with age-related differences [15]. For example, research has suggested that younger and older workers might be motivated by different aspects (e.g., younger by financial incentives or opportunities for learning new skills, while older by organizational pride and opportunities for applying their expertise [9,16,17]) and the adoption of practices overlooking these differences may increase the risk for intergenerational conflict.

For the purpose of preventing these dynamics, it is imperative for organizations to support age-friendly work environments. One way to achieve this is for organizations to address the age diversity climate [1]. This concept reflects employees’ perceptions of the extent to which organizational policies, practices, and procedures communicate organizational efforts towards supporting and valuing age diversity [18]. Therefore, a positive age diversity climate has been suggested to reduce the detrimental effect of self-categorization and the influence of age discrimination, while increasing the beneficial aspects associated with age diversity [1]. For example, an organization undertaking active steps to recruit and retain employees of all ages will be perceived by their employees as just and supportive, and perceptions of unfair treatment by one or more age groups will be less likely to emerge. As suggested by social exchange theory [19,20], a high-diversity climate is also likely to promote employees’ perceptions of being cared for by their organization, regardless of age [18]. As a result, employees will be willing to reciprocate through increased trust, higher performance and organizational citizenship behaviors, and reduced turnover [8,14,20,21,22]. 

While research investigating the effect of age diversity climate on organizational-level work-related outcomes (e.g., firm performance, organizational commitment) has been gaining prominence (e.g., [8,18]), less attention has been devoted to its impact on individual-level work-related outcomes (e.g., work engagement and wellbeing). 

In order to fill this gap, we adopted the social identity approach [11,12,13] and social exchange theory [19,20] as theoretical support in our investigation of the effect of age diversity climate (i.e., assessed as psychological climate) [21]) on individual-level work-related outcomes (i.e., behavioral, motivational, and wellbeing aspects of the employees in the workplace). Specifically, we investigated whether the age diversity climate affects intentions to quit, work engagement, and job-related wellbeing over and above individual (i.e., age, job tenure, perceived job- and organization-related fit) and contextual variables (i.e., job demands, job control, role clarity, and perceived supervisor support).

### 1.1. Social Identity Approach and Social Exchange Theory in Understanding Age Diversity Climate

The social identity approach [11,12,13] integrates both social identity [11] and self-categorization [13] theories and posits that individuals’ inclinations to internalize their membership to specific groups (e.g., being an older workers) based on salient categories (e.g., age) influence their behaviors, affect, and cognition towards the members of other groups (e.g., younger workers) [11,23]. This internalization of one’s membership triggers self-categorization processes, promoting a juxtaposition between “us” (i.e., ingroup; e.g., older workers) and “them” (i.e., outgroup; e.g., younger workers) based on that same salient characteristic [1,13,24]. This comparison between ingroup and outgroup can negatively affect workplace relationships by eliciting patterns of prejudice and discrimination, ultimately leading to negative organizational (e.g., increased workplace conflict, higher absenteeism) and individual outcomes (e.g., reduced wellbeing) [1,23]. 

The rising age diversity within workplaces is likely to increase the salience of age for self-identity processes [10]. This, in turn, is likely to increase age-based discrimination. For example, Kunze, Böhm and Bruch [10,14] found a significant positive relationship between age diversity and age discrimination climate (i.e., perceptions that the organizations’ procedures and practices are unfair towards individuals of different age [10]). Age discrimination has been linked with several negative outcomes for both the organization (e.g., performance decrease, increased retirement intentions) and the individual (e.g., higher depressive symptoms, lower job satisfaction and work engagement) [5,6,7]. 

These findings make a compelling case for why organizations should value age diversity and establish fair practices and procedures for workers of all ages to support a positive age diversity climate [1,20,22,25]. In fact, the age diversity climate has been highlighted as a mechanism that links the benefits of age diversity and positive work outcomes [1]. 

Another important theory used to develop and understand the effects of age diversity climate within workplaces is social exchange theory [1,20,21]. According to this approach, individuals act through a rational cost and benefit decision-making process [20] and every relationship is bounded to mutual (or reciprocative) behaviors that, in turn, promote further investment in the aforementioned relationship [20,22]. Within organizations, different types of relationships can be established—such as the one between employees and their employing organization—and each of these relationships will influence workplace behaviors. A relevant indicator of the quality of the relationship is organizational support [19,26]. Employees perceiving high organizational support will evaluate their relationship with the organization accordingly and, on the basis of this evaluation, experience several favorable outcomes (e.g., increased trust, high performance, engagement in organizational citizenship behaviors, reduced turnover) [14,20,21]. 

Age diversity climate may be an expression of organizational support. In fact, as Böhm, Kunze and Bruch [18] suggest, a high-diversity climate is likely to promote employees’ perceptions that they are cared for by the organization, regardless of their age, which then encourages important aspects of a social exchange relationship (i.e., trust, long-term orientation, perceptions of fairness) [20]. Indeed, previous studies reported a positive significant relationship between age diversity climate and perceptions of social exchange [18]. Given today’s salience of age within workplaces, employees are likely to not only use this information for social identity and self-categorization processes, but also rationally evaluate their organization’s level of support and fairness. Therefore, it is important to promote a positive age diversity climate that can foster trust, perceived fairness, and perceived organizational support among employees of all age.

### 1.2. Age Diversity Climate and Work-Related Outcomes at the Individual Level

Research supports the positive organizational-level outcomes of age diversity climate, such as increased firm performance and organizational commitment (e.g., [14,18,24]). For instance, Profili, Sammarra and Innocenti [8], in a sample of 326 Italian employees, found that age diversity climate positively impacted organizational citizenship behaviors through the effect of affective commitment. Moreover, Böhm, Kunze and Bruch [18] observed a positive effect of age diversity climate on performance, suggesting that an important indicator of firm performance is how well employees can work together despite their age differences. However, less attention has been devoted to individual-level work-related outcomes of age diversity climate, such as behavioral (e.g., intentions to quit), motivational (e.g., work engagement), and wellbeing (e.g., job-related wellbeing) aspects. These are key outcomes of interest in work and organizational psychology, and they have been widely investigated in this field of research (e.g., [25,27,28]), since they cover three different target areas of interest of management practices and strategies (i.e., behavioral/performance-related, wellbeing-related, and motivational/attitudinal-related). 

Numerous studies suggest that different individual and contextual factors, such as chronological age [29,30], job tenure [31,32], perceived job and organizational fit [33], role clarity [34,35,36], job demands [37], job control [37,38,39,40], and perceived supervisor support [38,41,42,43,44] can affect individuals’ wellbeing, motivation, and behaviors at work. However, limited research has investigated the effect of age diversity climate on these aforementioned outcomes. Specifically, to our knowledge, only one recent cross-sectional study investigated the effect of age diversity climate on motivational outcomes. Sousa, Ramos and Carvalho [45] surveyed 232 Portuguese employees and found that age diversity climate was positively associated with work engagement, which led to older workers’ preferences for late retirement. 

Due to the paucity of studies examining the impact of age diversity climate on individual-level work-related outcomes (i.e., intentions to quit, work engagement, and job-related wellbeing), further investigations are needed [1,46]. Based on the social identity approach and on the social exchange theory, the present study aims to evaluate the role of age diversity climate as a key predictor of workers’ wellbeing, engagement, and turnover intentions. In particular, we expect age diversity climate will be negatively related to worker’s intentions to quit (H1), positively related to job-related affective wellbeing (H2), and positively related work engagement (H3), over and above individual (i.e., age, job tenure, perceived job and organization fit) and contextual (i.e., job demands, job control, role clarity, perceived supervisor support) theoretically relevant covariates.

## 2. Methods

### 2.1. Participants and Procedure

The respondents were 110 Italian employees (42 females) aged between 18 and 61 years old (*M* = 46.10, *SD* = 10.02). In total, 52.7% (*n* = 58) of the participants had an undergraduate degree, while 47.3% (*n* = 52) were graduated. Concerning job type, 10.9% (*n* = 12) of the participants were blue collars, 49.1% (*n* = 54) were white collars, and 25.5% (*n* = 28) worked as upper management. The remaining 14.5% (*n* = 16) of participants selected “Other Job Type”. The average job tenure of the respondents was 23.82 years (*SD* = 10.42).

Data were collected using a time-lagged design, through online questionnaires (Qualtrics platform) at two time points between January 2020 and February 2020, 2–3 weeks apart. The time-lagged design was selected to control for common method variance, in addition to other procedural strategies recommended by Podsakoff, MacKenzie, Lee, and Podsakoff [47] (e.g., clarifying to the participants that there are no right or wrong answers, instructing them to answer as honestly as possible, and protecting their anonymity). Survey responses were matched using a univocal code chosen by participants to maintain their anonymity. At Time 1 (T1), participants provided information on demographic characteristics (i.e., age, gender, education, job type, job tenure), person–job and person–organization fit, role clarity, job demands, job control, and perceived supervisor support and age diversity climate. At Time 2 (T2), respondents provided information on their job-related affective wellbeing, intentions to quit, and work engagement. All participants voluntarily agreed to participate in the surveys, and informed consent was obtained from all workers involved in the study.

### 2.2. Measures

*T1 Age Diversity Climate*. We used the 4-item survey by Böhm, Kunze and Bruch [18]. The scale measures employees’ perceptions of the extent to which HR practices, procedures, and managers’ actions communicate support and fairness of treatment to workers of all age. A sample item is “Our company makes it easy for people from diverse age groups to fit in and be accepted”. Responses to items are given on a seven-point Likert scale ranging from 1 (“strongly agree”) to 7 (“strongly disagree”). Cronbach’s α = 0.83, 95% CI (0.77, 0.87).

*T1 Control Variables*. Numerous studies suggest that different individual and contextual factors, such as chronological age [29,30], job tenure [31,32], perceived job and organizational fit [33], role clarity [34,35,36], job demands [37], job control [37,38,39,40], and perceived supervisor support [38,41,42,43,44] can affect individuals’ wellbeing, motivation, and behaviors at work. Therefore, these variables were used as covariates in our study. *Age* and *job tenure* were included since previous studies have observed their role in influencing individuals’ wellbeing and intentions to continue working [35,36,48]. Participants’ perceived fit with their job and organization have been suggested to predict increased work engagement and reduced intentions to quit [49,50]. Perceived fit was assessed using two single items from Giumetti and Raymark [51], respectively: “Describe your overall level of fit with the job position. That is, how well do the requirements and tasks of the job seem to match with your knowledge, skills, and abilities?” and “Describe your overall level of fit with this organization. That is, how well do the values, personality and/or goals of the organization seem to match with your values, personality, and/or goals?”. Both items were rated on a 10-point scale ranging from 1 (“Very poor match”) to 10 (“Very good match”). Additionally, we assessed role clarity, job demands, job control, and perceived supervisor support following studies highlighting the relevance of such variables for our outcomes of interest e.g., [30,40,41,48,52,53,54]). For instance, high job demands and low job control have been repeatedly associated with reduced workers’ wellbeing [54], while low role clarity seems to predict higher intentions to quit [52]. We used four items from the UK *Health and Safety Executive Management Standards Indicator Tool* [55], one for each dimension: *role* “I am clear as to what my duties and responsibilities are”; *demand* “I have unrealistic time pressures”; *control* “I have some say over the way I work”; *supervisor support* “I am supported through emotionally demanding work”. Items were rated from 1 (“never”) to 5 (“always”). A similar procedure has been supported by Fisher, Matthews, and Gibbons [56] that positively evaluated the content validity and reliability of comparable single-item measures applicable to organizational and occupational health research. 

*T2 Job-related Positive Affective Wellbeing*. We measured job-related positive affective wellbeing using three items from van Katwyk, Fox, Spector, and Kelloway [57,58] questionnaire (i.e., “My job made me feel cheerful”, “My job made me feel enthusiastic”, and “My job made me feel content”). Responses were given on a five-point frequency scale from 1 (“never”) to 5 (“very often”). Cronbach’s α = 0.85, 95% CI (0.79, 0.89).

*T2 Work Engagement*. Work engagement was assessed using 3 items from Balducci, Fraccaroli, and Schaufeli’s [59] Italian validated scale, measuring a positive and fulfilling psychological state associated with one’s work (i.e., “My job inspires me”, “When I get up in the morning, I feel like going to work”, and “I am immersed in my job”). Responses were given on a seven-point scale from 0 (“never”) to 6 (“every day”) Cronbach’s α = 0.76, 95% CI (0.66, 0.82). 

*T2 Intentions to Quit*. We used three items from Landau and Hammer [60] to measure individuals’ intentions to leave their organizations (i.e., “As soon as I can find a better job, I will leave my organization”, “I am seriously thinking about quitting my job”, and “I am actively looking for a job outside my organization”). Responses were given on a seven-point Likert scale from 1 (“strongly disagree”) to 7 (“strongly agree”). Cronbach’s α = 0.94, 95% CI (0.91, 0.95).

### 2.3. Data Analysis

All the analyses were run using IBM SPSS [61]. First, preliminary examinations of the data (i.e., descriptives, correlations, reliabilities) were carried out. We also tested for discriminant validity of our variables and examined the assumptions of linearity, homoscedasticity, normality, independence, and multicollinearity to ensure they were being met [61]. Then, we proceeded testing our hypotheses by running hierarchical multiple linear regressions for each outcome of interest. In the first step, we entered all the control variables (i.e., age, job tenure, person–job and person–organization fit, and role clarity, job demands, job control, and perceived supervisor support). In the second step, we entered the age diversity climate. We examined the *R*^2^ increase following the inclusion of age diversity climate, to determine statistical significance of the relationships between the age diversity climate and each of the three outcomes.

## 3. Results

Descriptive statistics, reliabilities, and correlations are reported in Table 1.

Table 2 shows the results of the hierarchical multiple regressions.

**Hypothesis** **1** **(H1).** *Predicted that age diversity climate would be negatively associated with intentions to quit, over and above the control variables.*

H1 was supported because we observed a significant negative relationship between age diversity climate and intentions to quit (*β* = −0.38, *p* = 0.03), and there was a 4% change in *R*^2^ associated with the inclusion of age diversity climate in the model, *F* (9, 100) = 2.05, ∆*R^2^* = 0.04, *p* = 0.04.

**Hypothesis** **2** **(H2).** *Predicted that age diversity climate would be positively associated with job-related positive affective wellbeing, over and above the control variables.*

Hypothesis H2 was supported because we observed a significant positive relationship between age diversity climate and positive affective wellbeing (*β* = 0.17, *p* = 0.03), and there was a 4% change in *R*^2^ associated with the inclusion of age diversity climate in the model, *F*(9, 100) = 2.64, ∆*R^2^* = 0.04, *p* = 0.03.

**Hypothesis** **3** **(H3).** *Predicted that age diversity climate would be positively associated with work engagement, over and above the control variables.*

The regression model was not statistically significant; thus, hypothesis H3 was not supported, even if the addition of age diversity climate on Step 2 significantly increased *R*^2^, *F*(9, 100) = 1.50, ∆*R^2^* = 0.04, *p* = 0.05.

## 4. Discussion

The purpose of this study was to evaluate the role of age diversity climate as a predictor of individuals’ job-related wellbeing, turnover intentions, and work engagement, compared to other individual (i.e., age, job tenure, perceived job and organization fit) and contextual variables (i.e., job demands, job control, role clarity, perceived supervisor support). We based our hypotheses on the social identity approach [11,12,13] and social exchange theory [1,20,21]. The former helps to describe the effects of unmanaged age diversity (i.e., internalization of one’s membership to a particular age group, which in turn creates a juxtaposition between “us” and “them” and foster workplace conflict and discrimination), whereas the latter contributes to explaining the mechanism through which a positive age diversity climate can buffer such effects of age diversity by eliciting positive reciprocative behaviors (i.e., perceptions of support by one’s organization encourage employees’ trust and investment in the employee–employer relationship) [19,20,22,26].

In general, the results generally confirmed our hypotheses (i.e., H1 and H2), showing that age diversity climate negatively predicted turnover intentions and positively predicted job-related wellbeing over and above individual and contextual variables. Moreover, the control variables age, job tenure, and person–job fit emerged as predictors of job-related wellbeing.

However, we cannot fully support the effect of age diversity climate on work engagement. Specifically, the main effect of age diversity climate on work engagement was close to the significance threshold (*p* = 0.05), but the model was not significant. Therefore, our results are not in line with Sousa, Ramos, and Carvalho [45] that found a positive effect of age diversity climate on work engagement for older Portuguese workers. A possible explanation may be that such effect is stronger for the older group. Therefore, we may not have observed a significant relationship because our participant age range was wider and our sample was relatively small, which could have limited our ability to capture the effect. We suggest that future studies further explore the relationship between age diversity climate and work engagement.

### 4.1. Theoretical Implications

With regard to the theoretical advancements, this study expands prior findings on age diversity climate, suggesting that employees’ perceptions of organizational practices are not only related to organizational-level outcomes (e.g., firm performance), but also to individual-level ones (i.e., job-related wellbeing and intentions to quit). In fact, to our knowledge, this study is the first to explore the incremental validity of age diversity climate over other predictors (i.e., age, job tenure, perceived job and organization fit, role clarity, job demands, job control, and perceived supervisor support), answering the call for more studies on the effect of age diversity climate on individual-level work-related outcomes. First, as suggested by the social identity approach, increasing age diversity within work environments is likely to trigger a juxtaposition between different age groups that can negatively impact people and organizations [1,10,13]. This study contributes to showing that organizational policies, practices, and procedures supporting and valuing age diversity reduce the negative effects of self-categorization processes, increasing job-related wellbeing and decreasing intentions to leave the organization. Second, as suggested by the social exchange theory, employees’ workplace behaviors are based on the evaluation of the quality of their relationship with the employing organization, expressed through organizational support [19,21,23]. Our results corroborate the idea that a positive age diversity climate is perceived by employees as an expression of high organizational support, thus encouraging employees to reciprocate by displaying positive behaviors, resulting in favorable work outcomes. Finally, our results support and add knowledge to the age management research field (i.e., the study of the set of Human Resources Management policies, practices, and procedures oriented towards removing age-related obstacles and creating an age-friendly environment [62]). Specifically, our results are in line with what was found by the European Centre for the Development of Vocational Training [63]. In particular, they reviewed outcomes of age management practices across organizations and found that organizations that communicated their support towards employees of all ages benefited in terms of lower turnover/exit intentions and greater employee satisfaction and wellbeing. Moreover, our results mirror the studies on age discrimination climate [10] (i.e., the opposite of the age diversity climate construct framed by Böhm, Kunze and Bruch [18]) that linked age discrimination with reduced employee work engagement [7], intentions to permanently exit the organization [5,7], and negative self-rated health [6].

### 4.2. Practical Implications

From a practical standpoint, our findings corroborate the importance of supporting age diversity through a variety of HRM strategies. As our results suggest, a positive age diversity climate promotes individuals’ wellbeing and decreases their intentions to leave the organization. Given the labor shortages being observed in Western countries, this study makes a compelling case to prioritize age diversity climate in order to maximize employee retention [64]. Due to the ongoing demographic shift of an aging workforce, HR managers should be aware of the aspects that can impact employees’ decisions and motivation to continue working and support them in this endeavor. To promote even higher levels of age diversity, we support Böhm, Kunze and Bruch [18] suggestion for organizations and HR managers to not only implement age-inclusive HR practices, but also to actively highlight them in the hopes of promoting widespread awareness.

### 4.3. Limitations and Future Research

Although this study presented some conceptual and methodological strengths, the findings’ interpretation is bounded to some limitations. First, the online data collection led to a heterogeneous convenience sample, particularly in terms of jobs and organizations. Future studies may collect data on specific organizations, allowing a comparison between different sectors, which can help with supporting the generalizability of our findings. Despite the data being collected at two time points, 2–3 weeks apart, the absence of repeated measures limited our ability to account for the cross-lagged stability of the measured variables. Nonetheless, the time lag in the data collection constitutes a strength that can help reduce common method variance [47]. Future longitudinal studies might find it worthwhile to collect all measures at two or more time points and to verify possible changes in the scores. Additionally, future studies should test possible moderators and mediators of the age diversity climate’s relationship with individual-level outcomes. For instance, how age diversity climate is related to occupational future time perspective [65] or work ability [66] and how these affect wellbeing, motivation, and turnover intentions. Moreover, more focus on investigating age diversity climate antecedents (i.e., HR practices) should be considered because this could provide information as to how to develop positive age diversity climates. Additionally, examining new potential antecedents such as transformational leadership (i.e., considering the evidence supporting leadership style as a key enabler of positive diversity outcomes; [67]) could be a fruitful line of future research.

## 5. Conclusions

To conclude, this study contributes to existing literature addressing the need for more studies on the role of age diversity climate for individual-level work-related outcomes, such as turnover intentions, job-related wellbeing, and work engagement. Specifically, our findings support that age diversity climate affects workers’ intentions to quit and job-related affective wellbeing over and above individual (i.e., age, job tenure, perceived job and organization fit) and contextual variables (i.e., job demands, job control, role clarity, perceived supervisor support). This contribution reinforces the need for organizations to move towards age-friendly and age-inclusive workplaces.

## Figures and Tables

**Table 1:** Means, standard deviations, and intercorrelations among study variables (*n* = 110).

	Variables	*M*	SD	1	2	3	4	5	6	7	8	9	10	11	12
1.	T1 Age	46.10	10.02	-											
2.	T1 Job tenure	23.83	10.42	0.94 **	-										
3.	T1 Person–job fit	6.55	2.44	0.10	0.13	-									
4.	T1 Person–organization fit	5.54	2.41	−0.07	−0.03	0.60 **	-								
5.	T1 Role	4.27	0.88	−0.02	−0.02	0.14	0.29 **	-							
6.	T1 Demands	2.44	1.05	0.08	−0.05	−0.05	−0.08	−0.11	-						
7.	T1 Control	3.65	1.08	0.05	0.11	0.16	0.21 *	0.27 **	−0.15	-					
8.	T1 Support	2.90	1.17	−0.03	0.01	0.27 **	0.44 **	0.24 *	−0.20 *	0.23 *	-				
9.	T1 Age diversity climate	3.91	1.55	−0.10	−0.09	0.42 **	0.52 **	0.31 **	−0.28 **	0.32 **	0.51 **	(0.83)			
10.	T2 Intentions to quit	4.54	2.24	−0.04	−0.09	−0.11	−0.07	−0.19 *	0.13	−0.23 *	−0.21 *	−0.29 **	(0.94)		
11.	T2 Job-related positive affective wellbeing	3.11	1.02	0.01	0.11	0.24 *	0.06	0.03	−0.09	0.19 *	0.04	0.23 *	−0.51 **	(0.85)	
12.	T2 Work engagement	5.69	1.65	−0.10	−0.04	0.09	0.05	0.03	0.09	0.18	0.12	0.20 *	−0.47 **	0.70 **	(0.76)

Note: Cronbach’s alpha in brackets on the diagonal. * *p* < 0.05; ** *p* < 0.01.

**Table 2:** Results of the hierarchical multiple regression analysis (*n* = 110).

Heading	T2 Intentions To Quit										
Step/Variable	*F*	*R^2^*	*∆R^2^*	*B*	*SE*	*β*	*p*	*B*	*SE*	*β*	*p*
Step 1 (control variables)	1.69	0.11	0.11								
T1 Age				0.05	0.06	0.26	0.33	0.07	0.06	0.32	0.23
T1 Job tenure				−0.06	0.05	−0.31	0.25	−0.08	0.05	−0.39	0.14
T1 Person–job fit				−0.08	0.10	−0.09	0.44	−0.04	0.10	−0.04	0.71
T1 Person–organization fit				0.12	0.12	0.13	0.29	0.18	0.12	0.19	0.13
T1 Role				−0.34	0.25	−0.13	0.19	−0.28	0.25	−0.11	0.27
T1 Demands				0.13	0.20	0.06	0.50	0.04	0.20	0.02	0.83
T1 Control				−0.29	0.21	−0.14	0.15	−0.21	0.21	−0.10	0.32
T1 Support				−0.30	0.20	−0.16	0.14	−0.16	0.21	−0.08	0.45
Step 2	2.05 *	0.15	0.03 *								
T1 Age diversity climate								−0.38	0.18	−0.26	0.03
**Step/Variable**	**T2 Job-related positive affective wellbeing**										
	***F***	***R^2^***	***∆R^2^***	***B***	***SE***	***β***	***p***	***B***	***SE***	***β***	***p***
Step 1 (control variables)	2.27 *	0.15	0.15 *								
T1 Age				−0.06	0.02	−0.63	0.01	−0.07	0.02	−0.70	0.01
T1 Job tenure				0.06	0.02	0.64	0.01	0.07	0.02	0.73	0.01
T1 Person–job fit				0.12	0.04	0.30	0.01	0.10	0.04	0.25	0.03
T1 Person–organization fit				−0.06	0.05	−0.16	0.20	−0.09	0.05	−0.22	0.08
T1 Role				−0.001	0.11	0.001	0.99	−0.02	0.11	−0.02	0.80
T1 Demands				−0.05	0.09	−0.05	0.56	−0.01	0.09	−0.01	0.91
T1 Control				0.12	0.09	0.13	0.17	0.08	0.09	0.09	0.35
T1 Support				−0.03	0.09	−0.03	0.72	−0.10	0.09	−0.11	0.29
Step 2	2.64 **	0.19	0.03 *								
T1 Age diversity climate								0.17	0.08	0.27	0.03
**Step/Variable**	**T2 Work engagement**										
	***F***	***R^2^***	***∆R^2^***	***B***	***SE***	***β***	***p***	***B***	***SE***	***β***	***p***
Step 1 (control variables)	1.18	0.08	0.08								
T1 Age				−0.06	0.04	−0.40	0.14	−0.07	0.04	−0.46	0.09
T1 Job tenure				0.04	0.04	0.29	0.29	0.06	0.04	0.37	0.17
T1 Person–job fit				0.06	0.08	0.10	0.39	0.03	0.08	0.05	0.64
T1 Person–organization fit				−0.07	0.09	−0.10	0.42	−0.11	0.09	−0.16	0.22
T1 Role				−0.01	0.19	−0.01	0.94	−0.05	0.19	−0.03	0.77
T1 Demands				0.22	0.15	0.14	0.13	0.29	0.15	0.18	0.06
T1 Control				0.25	0.15	0.16	0.10	0.19	0.15	0.12	0.21
T1 Support				0.16	0.15	0.11	0.30	0.05	0.16	0.04	0.71
Step 2	1.50	0.12	0.04								
T1 Age diversity climate								0.26	0.13	0.25	0.05

Note: * *p* < 0.05; ** *p* < 0.01

## Data Availability

The data presented in this study are available on request from the corresponding author. The data are not publicly available due to privacy restrictions.
